# Coding-complete sequence of a vaccine-derived recombinant porcine reproductive and respiratory syndrome virus strain isolated in Hungary

**DOI:** 10.1007/s00705-019-04338-2

**Published:** 2019-07-12

**Authors:** S. Marton, D. Szalay, S. Kecskeméti, B. Forró, F. Olasz, Z. Zádori, I. Szabó, T. Molnár, K. Bányai, Á. Bálint

**Affiliations:** 1 Institute for Veterinary Medical Research, Centre for Agricultural Research, Hungarian Academy of Sciences, Hungária krt. 21, Budapest, 1143 Hungary; 2 Veterinary Diagnostic Directorate, National Food Chain Safety Office, Tábornok u. 2, Budapest, 1149 Hungary; 3 National Food Chain Safety Office, Budapest, Hungary

## Abstract

Porcine reproductive and respiratory syndrome virus 1 is a major cause of swine morbidity and mortality in various parts of the world, including Hungary. A national elimination programme to reduce the associated economic burden was initiated in Hungary in 2012. Using extensive laboratory surveillance, we identified and isolated an unusual PRRSV strain. The complete coding sequence of this isolate was determined and analyzed. The genome of this Hungarian PRRSV1 strain, HUN60077/16, is 15,081 nucleotides in length. Phylogenetic and recombination analysis showed a mosaic structure of the genome where a large fragment of ORF1b and the genomic region coding for ORF3 to ORF7 showed a very close genetic relationship to the vaccine virus Unistrain, while the ORF1a region, the 3’ end of ORF1b, and the whole ORF2 were only distantly related to this or any other PRRSV1 strain whose genome sequence is available in the GenBank database. Genomic characterization of PRRSV strains is crucial when possible vaccine-associated cases are identified. This approach not only helps to identify genetic interactions between vaccine and wild-type PRRSV1 strains but may also be needed to prevent trust in commercial vaccines from being undermined.

Porcine reproductive and respiratory syndrome (PRRS) is associated with severe economic losses to the swine industry worldwide. Clinically, PRRS in pregnant sows is characterized by reproductive disorders, including abortion, stillbirth and mummification, whereas in suckling and weaned piglets, pneumonia is a commonly observed manifestation [1].

The causative virus, porcine reproductive and respiratory syndrome virus (PRRSV), is a member of family *Arteriviridae*. It is a medium-sized enveloped virus with a positive-sense, single-stranded RNA genome of ~15 kb in length with at least 10 open reading frames (ORFs) [2]. The ORFs of PRRSV encode 16 non-structural proteins and eight structural proteins. Two large ORFs (ORF1a and ORF1b) that are located at the 5’ end and in the central region encode nsp1α, nsp1β, nsp2 to nsp6 (including nsp2TF and nsp2N), nsp7α, nsp7β, and nsp8 to nsp12 [2, 3]. ORFs 2a to 4 code for the membrane-associated glycoproteins GP2, GP3, and GP4 and a nonglycosylated membrane polypeptide, protein E. ORFs 5 to 7 encode three major structural proteins. These are, respectively, the envelope glycoprotein GP5, the nonglycosylated membrane protein M, and the nucleocapsid protein N, while two alternative ORFs overlapping with ORF5 and ORF7 code for the ORF5a protein and the 7ap protein, respectively [2–5].

PRRSVs are classified into two species, *Betaarterivirus suid 1* (former European genotype or genotype I; PRRSV1) and *Betaarterivirus suid 2* (former North American genotype or genotype II; PRRSV2) [6, 7]. The members of these two species differ from each other by >40% at the genome sequence level [7]. Vaccines have been developed against members of both species [8]. In Europe, where PRRSV1 is more prevalent, homotypic vaccine strains are currently used to alleviate the economic burden associated with PRRS. In Hungary, three live attenuated parenteral vaccines are used in swine herds. These are Porcilis (Merck Sharp and Dhome), Amervac/Unistrain (Hypra), and PRRSFlex (Boehringer).

Due to the economic burden of PRRS to the pig industry, Hungarian policymakers decided in 2012 to implement a national PRRS elimination programme. The programme is supported by continuous clinical and epidemiologic monitoring, which is supplemented by extended laboratory testing. Laboratory diagnosis is based on the detection of antibodies to PRRSV using a commercial enzyme immunoassay (INgezim PRRS Universal, Ingenasa), while detection of the genomic RNA is performed using a commercial real-time RT-PCR kit (Virotype PRRSV RT-PCR Kit, QIAGEN), and sequencing of amplified ORF5 and/or ORF7 is carried out by using published protocols [9].

In November 2016, laboratory tests identified the presence of PRRS in a herd in which the disease had previously been eliminated. The identified PRRSV strain was genetically closely related to the vaccine strain Unistrain (nt sequence identity: ORF5, 98.5%; ORF7, 97.4%), suggesting that it might have been a vaccine-derived PRRSV strain. These vaccine-derived PRRSV sequences were identified in clinically manifest infections characterized by sporadic occurrence of pregnant sows aborting and dying shortly thereafter. After laboratory diagnosis of PRRS in the herd, increased mortality among piglets and marked weight loss among weaned pigs was observed. The situation improved and became normal again two months after the first case was diagnosed. Although the outbreak was relatively mild, the clinical syndrome, together with available partial sequence data, prompted the authorities to seek further genetic information about the causative strain and its pathogenicity.

In this study, we report the coding-complete sequence of the PRRSV strain HUN60077/16, isolated from the disease outbreak.

The virus was isolated from a serum specimen on porcine alveolar macrophage cells. Genomic characterization started with random primed amplification of the genomic RNA derived from the cell-culture-isolated strain as described elsewhere [10, 11]. Sequencing was done on a 316 chip using Ion Torrent semiconductor sequencing equipment (Ion Torrent Personal Genome Machine; Life Technologies) according to the manufacturer’s recommendations. Sequences were assembled and aligned using the CLC Genomic Workbench v7.0 software (https://www.qiagenbioinformatics.com/), using a combination of *de novo* assembly and reference mapping as outlined earlier. A 15,081-base-long consensus sequence was obtained and deposited in the GenBank database under the accession number MK167464.

Molecular characterization of the coding-complete sequence of strain HUN60077/16 included annotation, phylogenetic analysis, and recombination analysis. The entire sequence was analyzed using the BLAST engine to find best hits and select additional sequences for further analysis. Of interest, the genome sequence of strain HUN60077/16 showed the highest whole-genome sequence similarity to the Unistrain strain (92% identity). The genome of HUN60077/16 was predicted to contain 13 ORFs. An overview of the coding potential is presented in Fig. 1. Eight major ORFs were compared individually to PRRSV sequences available in the GenBank database. Of interest, the genomic regions encoding ORF1b and ORF3 to ORF7 were highly similar to the corresponding genomic regions of the vaccine strain Unistrain. In contrast, the genomic regions coding for ORF1a and ORF2 were not closely related to Unistrain; moreover, no highly similar sequences from within this genomic region (Fig. 2a) were identified in GenBank.

**Fig. 1:**
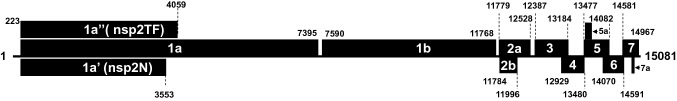
Schematic illustration of the genomic structure of the PRRSV1 strain HUN60077/16

**Fig. 2:**
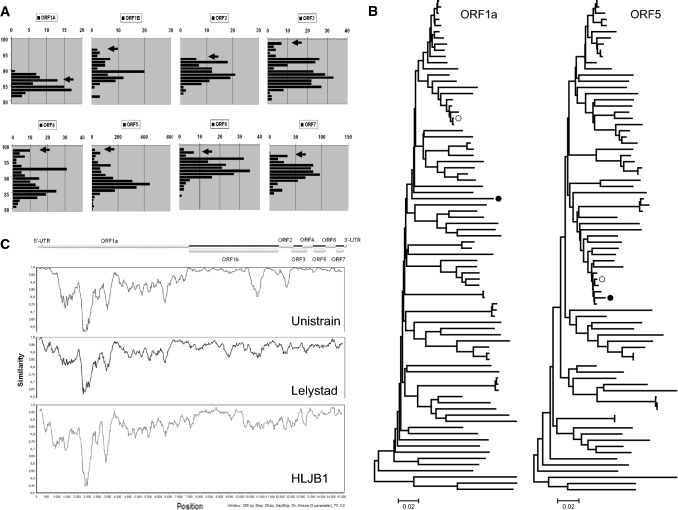
Panel A. The relative frequency of nt sequences of each ORF of selected PRRSV1 sequences with a given percentage of sequence identity to HUN60077/16 is compared to a representative number of. *y*-axis, percent sequence identity (range, 80 to 100%), *x*-axis, the number of sequences with the indicated level of sequence identity. The arrowhead indicates the level of sequence identity between the Hungarian strain and the Unistrain vaccine virus. B. Phylogenetic tree of representative genomic regions (neighbor-joining method, p-distance model). A black circle and a white circle designate the strains HUN60077/16 and Unistrain, respectively. The scale bar is proportional to the genetic distance. C. SimPlot analysis of HUN60077/16 using the Unistrain and Lelystad vaccine strains and a Chinese Amervac/Unistrain-derived recombinant field PRRSV1 strain

Phylogenetic analysis was performed using a set of sequences available in GenBank, and in our database, which contained >1000 ORF5 sequences. To illustrate the ambiguous position and phylogenetic relationship of HUN60077/16 to Unistrain and other reference strains we used the alignments of ORF1a region and the ORF5 [12, 13]. Overall, phylogenetic analysis of different genomic regions was consistent with the possibility that strain HUN60077/16 could have evolved through recombination between an Unistrain-derived strain and another, yet unidentified, PRRSV strain (Fig. 2b).

To confirm this hypothesis, we utilized the algorithms (RDP37, GENECONV38, MaxChi39, Bootscan/Recscan39, SiScan40, and 3Seq41) implemented in software RDP4 [14]. In addition, a sliding-window analysis tool, SimPlot, was utilized to visualize the putative recombination breakpoints [15]. In this analysis, we downloaded 80 nearly complete genome sequences of PRRSV1 strains from GenBank and identified Unistrain as a likely parental strain, donating a large fragment of ORF1b and the 3’ end region of the genomic RNA coding for ORF3 to ORF7. However, we failed to detect the other parental strain that donated ORF1a and the genomic region encompassing the 3’ end of ORF1b and the whole ORF2. Although the putative parental strain that donated these protein coding regions could not be determined by this approach using the available limited data set, our findings of a probable recombination event were confirmed using seven recombination detection algorithms as well as Simplot analysis (Fig. 2c).

Previous studies have indicated that PRRSV is prone to genomic recombination [16–20]; however, to the best of our knowledge, only one report from China describes a putative recombination event between Amervac and a field strain. This Chinese Amervac-derived recombinant strain [20], designated HLJB1, is unrelated to the Hungarian Unistrain-derived recombinant strain (Fig. 2c). From an epidemiological standpoint, it seems relevant to note that the recombination event in the Hungarian strain could have occurred some time ago, as the sequence identity in the genomic region most similar to the Unistrain-derived parental strain is below 99%, suggesting that some sequence variation occurred within the homologous genomic region before the detection of strain HUN60077/16.

Countries or even individual swine farms considering the implementation of a PPRSV elimination/eradication programme need to take into account the importance of genomic characterization of PRRSV strains to differentiate vaccine strains from wild-type strains. This is crucial for monitoring evolutionary mechanisms of field and vaccine strains and for preventing trust in commercial PRRS vaccines from being undermined due to the use of partial genome sequences for laboratory diagnosis and surveillance of PRRS.
